# A General FEM Model for Analysis of Third-Order Nonlinearity in RF Surface Acoustic Wave Devices Based on Perturbation Theory

**DOI:** 10.3390/mi13071116

**Published:** 2022-07-15

**Authors:** Baichuan Li, Qiaozhen Zhang, Xiangyong Zhao, Shaotao Zhi, Luyan Qiu, Sulei Fu, Weibiao Wang

**Affiliations:** 1 College of Information, Mechanical and Electrical Engineering, Shanghai Normal University, Shanghai 200234, China; 1000494740@smail.shnu.edu.cn (B.L.); zhist@shnu.edu.cn (S.Z.); 2 Key Laboratory of Optoelectronic Material and Device, Department of Physics, Shanghai Normal University, Shanghai 200234, China; xyzhao@shnu.edu.cn; 3 The School of Materials Science and Engineering, Tsinghua University, Beijing 100084, China; 4 Shoulder Electronics Limited, Wuxi 214124, China; wangweibiao@wxsde.cn

**Keywords:** surface acoustic wave (SAW), perturbation theory, finite element method (FEM), nonlinearity, generation mechanisms

## Abstract

This article presents a general-purpose model that enables efficient and accurate calculation of third-order nonlinear signals in surface acoustic wave (SAW) devices. This model is based on piezoelectric constitutive equations combined with perturbation theory, which can be analyzed by full finite element method (FEM). For validation, third-order harmonic (H3) responses and intermodulation distortions (IMD3) in SAW resonators are simulated, and their calculation results fit well to experimental data in the literature. Then, the generation mechanisms of the third-order nonlinearity in SAW resonators are discussed. The dominant generation mechanisms for different nonlinear signals and the relation between electrode materials and H3 peak magnitude are revealed, which provides an important guideline for further nonlinear suppression.

## 1. Introduction

Surface acoustic wave and bulk acoustic wave (SAW/BAW) devices used as radio frequency (RF) front-end filters and duplexers are mass-produced and applied in telecommunication systems [1,2]. Recent advances in mobile communication technologies have led to multiple signal handling, downsizing, and higher power level operations [3]. In this circumstance, the nonlinearity in SAW/BAW has become an important issue, as it generates harmonic and intermodulation distortion (IMD) products, which result in noticeable signal distortions [4,5,6].

Numerous groups have made efforts to investigate the nonlinearity in SAW/BAW devices and its suppression. For BAW devices, the one-dimensional (1D) Mason equivalent circuit model and modified Butterworth Van Dyke (MBVD) model are commonly employed [3,7,8]. Shim and Feld [9] proposed a 1D nonlinear Mason model using the harmonic balance (HB) technique [10,11]. The proposed Mason model is applicable to arbitrary piezoelectric nonlinear sources, and it simulates well for nonlinear signals generated in RF BAW. Considering the nonlinearity in RF BAW devices is extremely weak, Hashimoto et al. [12,13] pointed out that the perturbation method should be more applicable than the HB method. They demonstrated that the nonlinear behaviors of the thickness extensional resonators for both 1D and 2D cases are accurately simulated by the MBVD model with the first-order perturbation method. Chen et al. [14,15] also applied the above method to calculate third-order nonlinear distortion of SAW duplexers. The validity and accuracy were established by comparing simulation and measurement results.

As for SAW devices, the classical coupling-of-modes (COM) and P-matrix model methods are widely used to characterize their nonlinear behaviors. Nakagawa et al. [16] derived a COM model by introducing the nonlinear stress and electric displacement to identify the contributions from different mechanisms to nonlinearity in SAW devices. Furthermore, Chauhan et al. [17,18] extended it to P-matrix formalism to analyze that of the temperature compensated SAW (TC-SAW) devices on 128° YX LiNbO_3_ substrate. In the latest publications, a finite element method (FEM) model was applied by Mayer et al. [19], Guan et al. [20] and Pang et al. [21] to study the intermodulation and harmonic generation in LiNbO_3_ based SAW resonators. However, the above nonlinear FEM models are limited to SAW resonators on piezoelectric substrate such as Quartz and LiNbO_3_, whose higher-order material constants are known. This means they have difficulty in analyzing the cases when higher-order material constants are unknown, for example, the commercial commonly used LiTaO_3_.

With the coming of the 5G era, SAW/BAW and hybrid SAW-BAW devices with complicated structures are emerging one after another. Although the above publications have illustrated the generation mechanisms of nonlinearity and proposed several nonlinearity suppression methods, a general-purpose and efficient simulation tool for both SAW and BAW devices is still absent. In our previous work [22], we proposed a full FEM model for analyzing nonlinearity in BAW resonators and verified its effectiveness. Therefore, in this paper, we extend this model to calculate nonlinearity in SAW resonators. Theoretical derivations of this method are based on piezoelectric constitutive equations and perturbation theory, by which multiple piezoelectric nonlinearity can be considered simultaneously and combined arbitrarily. Compared to the previous FEM model [19,20,21], the advantage of the proposed model is that it not only inherently has the universality of a finite element, but also remains effective even if higher-order material constants of piezoelectric substrate are unknown. Validations were made by comparing the third-order nonlinear responses obtained by the proposed method with experimental results in the literature qualitatively. In addition, possible nonlinearity generation mechanisms and their suppression are discussed.

## 2. Theoretical Background and Analysis Procedures

### 2.1. Linear Equations of Piezoelectricity

We start by linear cases for piezoelectric resonators, the linear constitutive equations with charge-stress form (namely *e*-form) describe piezoelectric coupling as follows:

(1) $$T=c^{E}S-eE$$

And

(2) $$D=eS+\varepsilon^{S}E$$

where *T*, *S*, *E* and *D* are the stress, strain, electric field and electric displacement, respectively, and $c^{E}$, e and $\varepsilon^{S}$ are the elastic stiffness constant under constant *E*, piezoelectric coefficient and dielectric constant under constant *S*, respectively. Note that the subscripts of variables and material constants were omitted to simplify the following derivation.

The motion of the piezoelectric body is governed by Newton’s second law:

(3) $$\nabla \;\cdot \;T=\rho \frac{\partial^{2}u}{\partial t^{2}}$$

where ρ is the mass density and u is displacement, and the charge equation of electrostatics is given by:

(4) ∇ · D=0

Therefore, the electric displacement D is spatially uniform.

### 2.2. Nonlinear Equations of Piezoelectricity

On the basis of the perturbation theory [23], we modify the linear piezoelectric constitutive equations by introducing the nonlinear stress $T_{N}$ and the nonlinear electric displacement $D_{N}$ perturbations as follows:

(5) $$T=c^{E}S-eE+T_{N}$$

And

(6) $$D=eS+\varepsilon^{S}E+D_{N}$$

where $T_{N}$ and $D_{N}$ can be represented as a series consisting of integer powers of their variables S and E.

According to the thermodynamics of solids [24], we derived the expression of perturbation terms $T_{N}$ and $D_{N}$ by expanding Helmholz free energy A until the fourth-order terms of strain S and electric field E:

(7) $$\begin{array}{cc} T_{N}= & \frac{1}{2!}\left(\frac{\partial^{3}A}{\partial S^{3}}\right)S^{2}+\left(\frac{\partial^{3}A}{\partial S^{2}\partial E}\right)SE+\frac{1}{2!}\left(\frac{\partial^{3}A}{\partial S\partial E^{2}}\right)E^{2}\\ + & \frac{1}{3!}\left(\frac{\partial^{4}A}{\partial S^{4}}\right)S^{3}+\frac{1}{2}\left(\frac{\partial^{4}A}{\partial S^{3}\partial E}\right)S^{2}E+\frac{1}{2}\left(\frac{\partial^{4}A}{\partial S^{2}\partial E^{2}}\right)SE^{2}+\frac{1}{3!}\left(\frac{\partial^{4}A}{\partial S\partial E^{3}}\right)E^{3} \end{array}$$

And

(8) $$\begin{array}{cc} D_{N}= & -\frac{1}{2!}\left(\frac{\partial^{3}A}{\partial S^{2}\partial E}\right)S^{2}-\left(\frac{\partial^{3}A}{\partial S\partial E^{2}}\right)SE-\frac{1}{2!}\left(\frac{\partial^{3}A}{\partial E^{3}}\right)E^{2}\\ - & \frac{1}{3!}\left(\frac{\partial^{4}A}{\partial S^{3}\partial E}\right)S^{3}-\frac{1}{2}\left(\frac{\partial^{4}A}{\partial S^{2}\partial E^{2}}\right)S^{2}E-\frac{1}{2}\left(\frac{\partial^{4}A}{\partial S\partial E^{3}}\right)SE^{2}-\frac{1}{3!}\left(\frac{\partial^{4}A}{\partial E^{4}}\right)E^{3} \end{array}$$

Equations (7) and (8) are then rewritten as Equations (9) and (10), respectively, as follows:

(9) $$\begin{array}{cc} T_{N}= & \frac{1}{2}\chi_{20}^{T}S^{2}+\chi_{11}^{T}SE+\frac{1}{2}\chi_{02}^{T}E^{2}\\ + & \frac{1}{6}\chi_{30}^{T}S^{3}+\frac{1}{2}\chi_{21}^{T}S^{2}E+\frac{1}{2}\chi_{12}^{T}SE^{2}+\frac{1}{6}\chi_{03}^{T}E^{3} \end{array}$$

And

(10) $$\begin{array}{cc} D_{N}= & -\frac{1}{2}\chi_{11}^{T}S^{2}-\chi_{02}^{T}SE-\frac{1}{2}\chi_{02}^{D}E^{2}\\ - & \frac{1}{6}\chi_{21}^{T}S^{3}-\frac{1}{2}\chi_{12}^{T}S^{2}E-\frac{1}{2}\chi_{03}^{T}SE^{2}-\frac{1}{6}\chi_{03}^{D}E^{3} \end{array}$$

where $\chi_{ij}^{T}$ and $\chi_{ij}^{D}$ are nonlinear coefficients, and the superscripts “*T*” and “*D*” indicate contributions of $T_{N}$ and $D_{N}$. Note that the dominated terms in $T_{N}$ and $D_{N}$ expressions are related to the order of nonlinearity under consideration.

Substituting Equations (5) and (6) into Equations (3) and (4) derives:

(11) $$\nabla \cdot \left(c^{E}S-eE\right)-\rho \frac{\partial^{2}u}{\partial t^{2}}=-\nabla \cdot T_{N}$$

And

(12) $$\nabla \cdot \left(eS+\varepsilon^{S}E\right)=-\nabla \cdot D_{N}$$

Nonlinear responses in the piezoelectric resonator can be determined by linear partial differential equations of Equations (11) and (12) where the terms on the right-hand side are taken as perturbations.

### 2.3. Derivation of Third-Order Nonlinear Responses

For simplicity, it is assumed that the excitation with two different frequency components $f_{1}$ and $f_{2}$ are applied to a piezoelectric resonator. Therefore, the linear strain and electric field in piezoelectric material are expressed as:

(13) $$S=S_{f_{1}}+S_{f_{2}}$$

And

(14) $$E=E_{f_{1}}+E_{f_{2}}$$

The perturbations caused by nonlinear products including intermodulation distortions and harmonics responses can be estimated by substituting Equations (13) and (14) into Equations (9) and (10). The nonlinear stress $T_{N}$ and nonlinear electric displacement $D_{N}$ for third-order harmonics (H3) are derived as:

(15) $$T_{N}^{3f_{1}}=\frac{1}{6}\chi_{30}^{T}S_{f_{1}}^{3}+\frac{1}{2}\chi_{21}^{T}S_{f_{1}}^{2}E_{f_{1}}+\frac{1}{2}\chi_{12}^{T}S_{f_{1}}E_{f_{1}}^{2}+\frac{1}{6}\chi_{03}^{T}E_{f_{1}}^{3}$$

And

(16) $$D_{N}^{3f_{1}}=-\frac{1}{6}\chi_{21}^{T}S_{f_{1}}^{3}-\frac{1}{2}\chi_{12}^{T}S_{f_{1}}^{2}E_{f_{1}}-\frac{1}{2}\chi_{03}^{T}S_{f_{1}}E_{f_{1}}^{2}-\frac{1}{6}\chi_{03}^{D}E_{f_{1}}^{3}$$

In a similar way, the expressions of $T_{N}$ and $D_{N}$ for third-order intermodulation distortions (IMD3) cases are derived as:

(17) $$\begin{array}{cc} T_{N}^{2f_{1}\pm f_{2}}= & \frac{1}{2}\chi_{30}^{T}S_{f_{1}}^{2}S_{f_{2}}\\ + & \frac{1}{2}\chi_{21}^{T}\left(2S_{f_{1}}E_{f_{1}}S_{f_{2}}+S_{f_{1}}^{2}E_{f_{2}}\right)\\ + & \frac{1}{2}\chi_{12}^{T}\left(2S_{f_{1}}E_{f_{1}}E_{f_{2}}+S_{f_{2}}E_{f_{1}}^{2}\right)\\ + & \frac{1}{2}\chi_{03}^{T}E_{f_{1}}^{2}E_{f_{2}} \end{array}$$

And

(18) $$\begin{array}{cc} D_{N}^{2f_{1}\pm f_{2}}= & -\frac{1}{2}\chi_{21}^{T}S_{f_{1}}^{2}S_{f_{2}}\\ - & \frac{1}{2}\chi_{12}^{T}\left(2S_{f_{1}}E_{f_{1}}S_{f_{2}}+S_{f_{1}}^{2}E_{f_{2}}\right)\\ - & \frac{1}{2}\chi_{03}^{T}\left(2S_{f_{1}}E_{f_{1}}E_{f_{2}}+S_{f_{2}}E_{f_{1}}^{2}\right)\\ - & \frac{1}{2}\chi_{03}^{D}E_{f_{1}}^{2}E_{f_{2}} \end{array}$$

It is noted that perturbation terms $T_{N}$ and $D_{N}$ in Equations (5) and (6) are composed by the combination of the electrostatic field, the strain field, and the mixing of the strain field and the electrostatic field [25], which can be selectively estimated by choosing specific expressions from Equations (15)–(18) for different nonlinear responses.

### 2.4. Analysis Procedures of Nonlinear Signals

**Figure 1:**
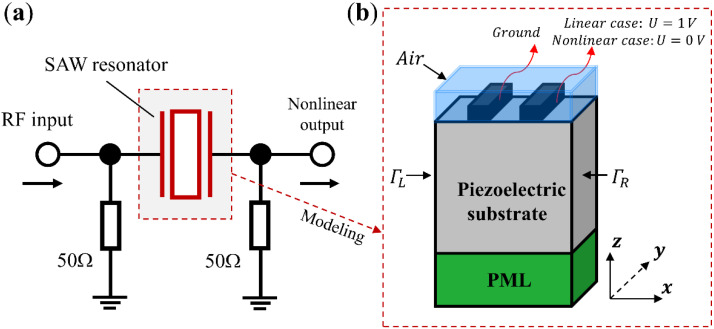
(**a**) Schematic of a SAW resonator with a peripheral circuit (**b**) quasi-3D model for the SAW resonator used in simulation. (not to scale).

Figure 1a shows a schematic of a SAW resonator under nonlinear test with 50 Ω matching impedance. The SAW resonator with an infinitely long interdigital transducers (IDTs) structure, comprised of periodic metal electrodes, is considered. For modeling of this device, a quasi-3D periodic FEM model (shown in Figure 1b) is built and used for numerical simulations by using a built-in piezoelectric module of FEM software COMSOL Multiphysics 5.6, as two-dimensional (2D) models are insufficient in analyzing the horizontal shear field component along the aperture y-direction. As shown, one period with periodic metal electrodes is considered, the continuity periodic boundary condition is applied to field variables at the left surface ($\Gamma_{L}$) and the right surface ($\Gamma_{R}$). For modeling accuracy, an air layer overlay is added to the top of the IDTs to consider the parallel capacitance effect. The perfectly matched layer (PML) is applied to the bottom to reduce the model size and suppress the unwanted boundary reflection. The periodic metal electrodes are applied with a terminal of one voltage and ground, respectively, for linear analysis, and then the terminal is changed to zero voltage for nonlinear analysis. As for the calculation of nonlinear responses, co-simulation of the quasi-3D FEM model of the resonator with its peripheral circuit is performed by using LiveLink of COMSOL with MATLAB.

**Figure 2:**
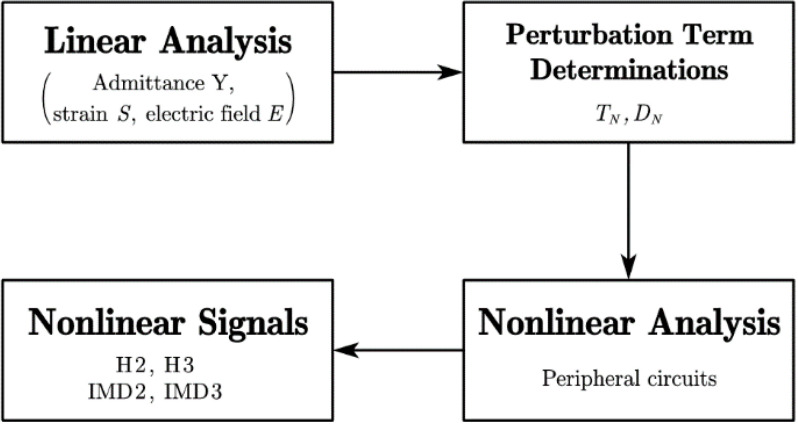
Analysis procedures for simulation of nonlinear signals.

The SAW resonator is characterized by the harmonic analysis, and the harmonic admittance Y per IDT period is estimated by Y=2πfjQ/U, where f is the driving frequency, U is the applied electric potential and Q is the total charge induced on the electrode. Figure 2 shows a flow chart illustrating the procedures for analysis of nonlinear responses. As shown, the linear input admittance $Y_{11}$ of the resonator at both driving frequency and output frequency is first evaluated by a quasi-3D FEM model using COMSOL. Meanwhile, the linear strain S and electric field E at same frequency spectrum are also obtained. Then, the nonlinear terms $T_{N}$ and $D_{N}$, combination terms of linear strain S and electric field E with nonlinear coefficients $\chi_{ij}$, are estimated and added into the linear model as perturbations. To be specific, $T_{N}$ and $D_{N}$ can be added as mechanical and electric loading into “solid mechanics” and “electrostatics” interfaces in COMSOL, respectively. Next, the effects of a peripheral circuit, such as die, package and matching impedance effects, are taken into account using LiveLink with a MATLAB interface. Finally, different nonlinear responses can be obtained by solving the nonlinear piezoelectric constitutive equations at an aiming output frequency range, provided that a good priori of nonlinear coefficients $\chi_{ij}$ are given. It is noted that priori values of nonlinear coefficients $\chi_{ij}$ are obtained by fitting simulation results with experimental results. In the fitting procedure, nonlinear coefficients $\chi_{ij}$ were tried one by one and optimized by minimizing the absolute difference between the simulated and measured results. Additionally, multiple nonlinear coefficients can be considered simultaneously to find the best agreement with the measurement.

## 3. Simulation Results and Validations Examples

**Figure 3:**
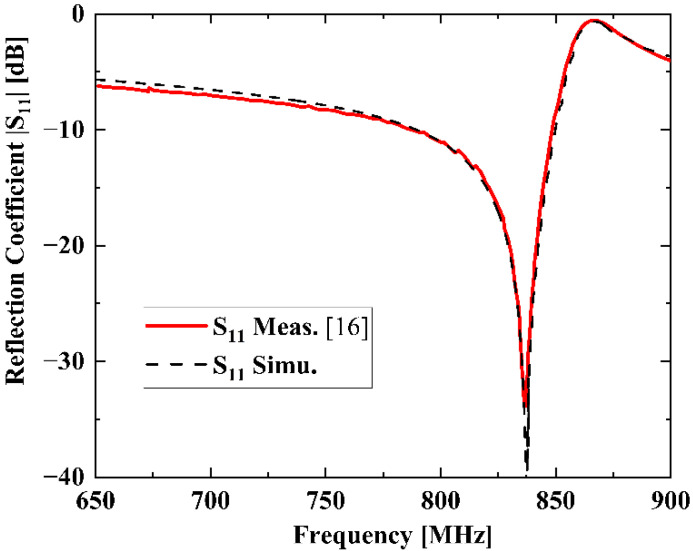
Measured and simulated reflection coefficient $S_{11}$ of the SAW resonator.

To confirm validity for SAW devices of the proposed model, a SAW resonator on 42° YX LiTaO_3_ (42-LT) substrate is taken as an example. Figure 3 shows the reflection coefficient $S_{11}$ of the 42-LT SAW resonator. As shown, the $S_{11}$ curve calculated by the quasi-3D FEM model fits fairly well with the measured results in [16]. A resonant frequency $f_{r}$ exists at 837.1 MHz and an anti-resonant frequency $f_{a}$ at 866.8 MHz.

**Figure 4:**
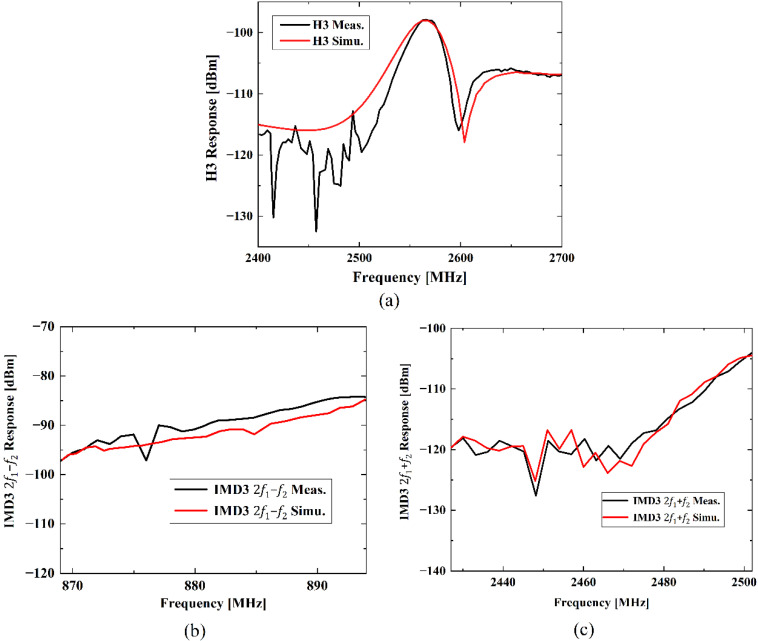
Measured [16] and simulated nonlinear responses of the SAW resonator: (**a**) H3 response, (**b**) IMD3 $2f_{1}-f_{2}$ response, (**c**) IMD3 $2f_{1}+f_{2}$ response.

For H3 simulation, a continuous wave (CW) signal was used and its incident power level is 15 dBm. The driving frequency is swept from 800 to 900 MHz, and an H3 signal appears at triple the driving frequency, namely from 2.4 to 2.7 GHz. As shown in Figure 4a, the calculated H3 frequency dependence also compares well with that of the experiment in [16], particularly, the peak and notch shape. A peak with the maximum H3 level at 2.56 GHz can be seen and a steep notch occurs at about 2.6 GHz. In this case, nonlinear parameters $\chi_{21}^{T}$, $\chi_{03}^{T}$ and $\chi_{03}^{D}$ in Equation (16) are used for calculation.

As for simulations of IMD3, two excitation CW signals with frequencies $f_{1}$ and $f_{2}$ are applied to the quasi-3D FEM model of the 42-LT SAW resonator. The incident power levels of the two input tones are 15 dBm as well. In this case, the driving frequency $f_{1}$ is swept from 824 to 849 MHz, and $f_{2}$ is equal to $f_{1}-45$ MHz. Thus, IMD3 $2f_{1}-f_{2}$ response can be found at 869 to 894 MHz and IMD3 $2f_{1}+f_{2}$ response will appear at 2427 to 2502 MHz. The simulated results of IMD3 signals fitted to each measured one are shown in Figure 4b,c, respectively. As shown, simulations of both the IMD3 signal with $2f_{1}-f_{2}$ and the IMD3 signal with $2f_{1}+f_{2}$ exhibit decent agreement with the measured results in [16]. In these simulations, $\chi_{30}^{T}$ in Equation (17) is used to predict the IMD3 $2f_{1}-f_{2}$ response, and $\chi_{21}^{T}$, $\chi_{03}^{T}$ in Equation (18) are applied for the calculation of IMD3 $2f_{1}+f_{2}$ response.

## 4. Generation Mechanisms and Suppression of Nonlinearity

The proposed model can study the contributions of different generation mechanisms to nonlinearity in SAW resonators by setting the corresponding nonlinear parameters $\chi_{ij}$ in Equations (9) and (10) to zero and non-zero. Thus, discussions on generation mechanisms of nonlinearity can be given based on simulation results of the above-mentioned SAW resonator on 42-LT substrate.

### 4.1. Generation Mechanisms of Third-Order Nonlinearity

Nonlinear effects in elasticity, dielectric, and electro-mechanical coupling such as piezoelectricity and electrostriction will contribute to nonlinear signals generated in SAW devices. To find the dominant contributions to third-order nonlinearity, nonlinear terms composed of different nonlinear coefficients $\chi_{ij}$ combined with strain S and electric field E were investigated, respectively. Linear strain and electric field components of $S_{yz}$ and $E_{z}$ were used as they are expected to be the predominant components for SH-type SAW. Similarly, $T_{xy}$ and $D_{z}$ were selected and added as perturbations.

**Figure 5:**
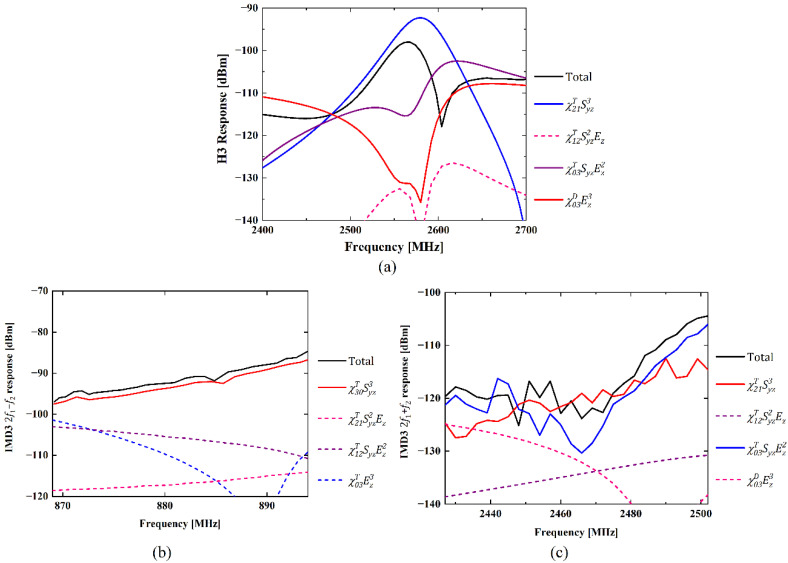
Contributions of employed nonlinear terms to different nonlinear responses: (**a**) H3 response, (**b**) IMD3 $2f_{1}-f_{2}$ response, (**c**) IMD3 $2f_{1}+f_{2}$ response.

Figure 5a–c illustrate the separate contributions of different nonlinear terms to the simulation results shown in Figure 4a–c, respectively. Perturbations in $D_{N}$ are considered for the H3 and IMD3 $2f_{1}+f_{2}$ cases as their output frequency $f\gg f_{r}$, and that in $T_{N}$ are used for the IMD3 $2f_{1}-f_{2}$ case as the output frequency is adjacent to the resonant frequency $f_{r}$. For the H3 simulation shown in Figure 5a, nonlinear terms $\chi_{21}^{T}S_{yz}^{3}$, $\chi_{03}^{T}S_{yz}E_{z}^{2}$ and $\chi_{03}^{D}E_{z}^{3}$ in $D_{z}$ are of significant contributions, whereas the effect of $\chi_{12}^{T}S_{yz}^{2}E_{z}$ is negligible. $\chi_{21}^{T}S_{yz}^{3}$ term contributes to a simple peak dependency of the H3 curve, and the notch at about 2.6 GHz in Figure 5a is mainly caused by the coupling effect of nonlinear terms $\chi_{21}^{T}S_{yz}^{3}$ and $\chi_{03}^{D}E_{z}^{3}$. Thus, nonlinear piezoelectricity and dielectric represented by $\chi_{21}^{T}S_{yz}^{3}$ and $\chi_{03}^{D}E_{z}^{3}$, respectively, are predominant for H3 generation. For the simulation of IMD3 $2f_{1}-f_{2}$ response shown in Figure 5b, only $\chi_{30}^{T}S_{yz}^{3}$ in $T_{xy}$ matters and the contributions from the other three nonlinear terms are insignificant. It means nonlinear elasticity induced by $\chi_{30}^{T}S_{yz}^{3}$ is responsible for the IMD3 $2f_{1}-f_{2}$ response in the SAW resonator. As for the simulation of the IMD3 $2f_{1}+f_{2}$ response shown in Figure 5c, $\chi_{21}^{T}S_{yz}^{3}$, $\chi_{03}^{T}S_{yz}E_{z}^{2}$ in $D_{z}$ are dominant in this case, and effects from the other two nonlinear terms can be neglected. Namely, nonlinear piezoelectricity caused by $\chi_{21}^{T}S_{yz}^{3}$ are also the major source of IMD3 $2f_{1}+f_{2}$ generation. It is concluded that the nonlinear effect of acoustic strain, i.e., $S^{3}$ terms in Equations (9) and (10), contributes to both H3 and IMD3 responses considerably. Therefore, nonlinear elasticity and piezoelectricity generated by acoustic strain are the dominant sources for third-order nonlinear responses of SAW devices.

### 4.2. Nonlinearity Suppression

In the last section, we point out that the third-order nonlinear signals of SAW devices are generated by nonlinear elasticity dominantly. Nakagawa et al. [26] also investigated the effect of different Ti layer thicknesses on H3 generation in SAW resonators with Al/Ti layered electrodes, which consist of metals with different elastic constants. Furthermore, they demonstrated that the H3 response of the SAW resonator decreases obviously with an increase in the thickness of the adhesive layer, Ti.

**Figure 6:**
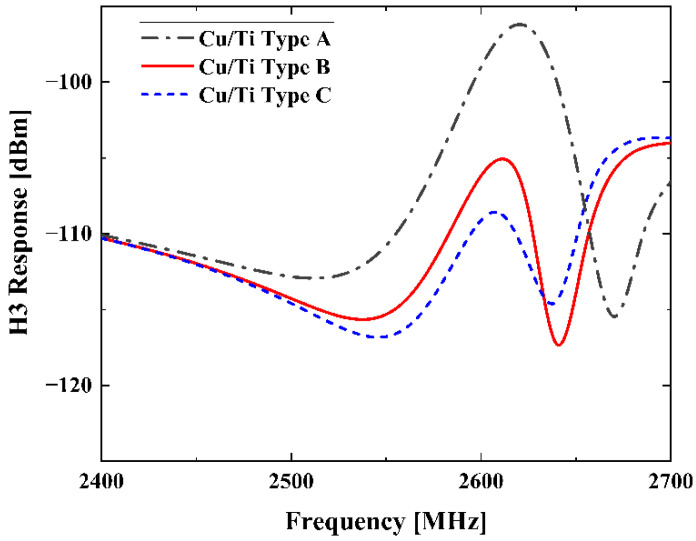
Simulated H3 responses of the SAW resonators in Table 1.

Similarly, we calculated the H3 signals of SAW resonators on 42-LT with three different Cu/Ti electrode structures, and the designed layer thicknesses are given in Table 1. As shown in Figure 6, the variation in H3 magnitude with Ti layer thickness is consistent with [26].

Furthermore, H3 calculation is performed for the SAW resonators with electrode materials of different Young’s modulus. Table 2 gives the parameters related to the elasticity of different electrode materials, where E is Young’s modulus, v is Poisson’s ratio, $c_{12}$ and $c_{44}$ are the two independent elastic constants for isotropic materials.

**Figure 7:**
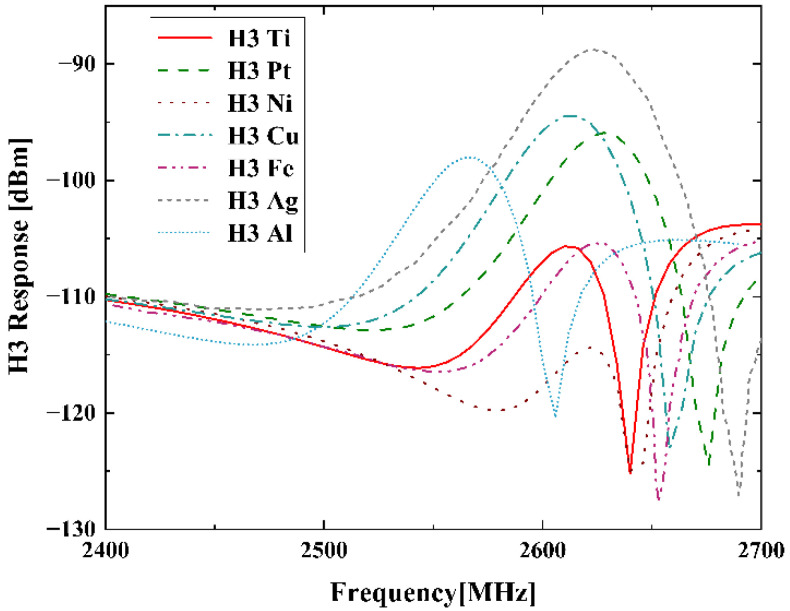
Simulated H3 responses of the SAW resonators with different electrode materials.

**Figure 8:**
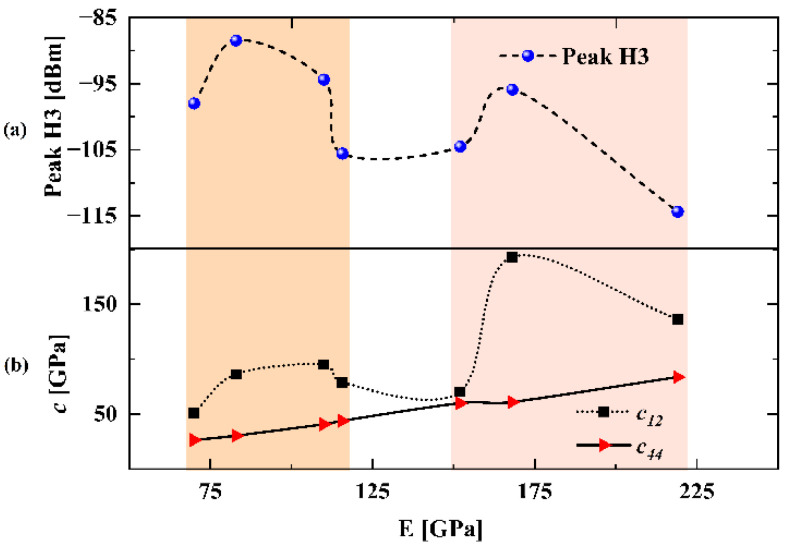
(**a**) Simulated H3 peak magnitude of the SAW resonators with different electrode materials. (**b**) Elastic constants of different electrode materials arranged in ascending order of Young’s modulus.

In simulations, thicknesses of different electrode materials are adjusted to keep the same resonant frequency $f_{r}$ ∼ 850 MHz. Figure 7 shows the simulated H3 results of the SAW resonators on 42-LT substrate with different electrode materials listed in Table 2. It is seen that the H3 curves in Figure 7 exhibit similar frequency dependence with those curves shown in the above Figure 4a. Then, Figure 8a compares the H3 peak values corresponding to different electrode materials in this calculation. These values are arranged in ascending order of Young’s modulus, and the values of $c_{12}$ and $c_{44}$ are organized in same order as well in Figure 8b. As shown, the H3 peak curve has the same trend as the $c_{12}$ curve, except for the values of $c_{44}$ being closer to or greater than $c_{12}$. This means the larger one of the two independent elastic constants $c_{12}$ and $c_{44}$ possesses a more dominant contribution for H3 generation. It should be noted that the H3 peak can be suppressed by about 25 dBm by choosing proper electrode materials, which provides a vital insight into improving the linearity of SAW devices.

## 5. Conclusions

In this paper, we proposed a general FEM model for analyzing third-order nonlinear signals in RF SAW resonators based on perturbation theory. For validation, simulations of the H3 response and IMD3 response for a SAW resonator on 42-LT substrate are performed. The comparison of simulation results with measured results in the literature demonstrated the accuracy of the proposed model. The generation mechanisms of third-order nonlinearity in SAW resonators are discussed in detail and the dominant mechanisms are distinguished to give a guideline for further nonlinear suppression. In particular, the relation between elastic constants of electrode materials and H3 peak value is concluded. Additionally, due to the generality of the proposed nonlinear FEM model, this model could be extended to analyze the nonlinearity of SAW/BAW devices based on arbitrary structure configurations and materials for further study.

## Figures and Tables

**Table 1:** Layer Thicknesses of Different Electrodes.

Electrode Type	Cu Thickness (nm)	Ti Thickness (nm)
A	136.5	15
B	100	87.6
C	80	127.3

**Table 2:** Parameters Related to the Elasticity of Different Electrode Materials.

Metal	E [GPa]	v	$c_{12}$ [GPa]	$c_{44}$ [GPa]
Al	70.00	0.33	51.08	26.32
Ag	83.00	0.37	86.22	30.29
Cu	110.00	0.35	95.06	40.74
Ti	115.70	0.32	78.53	43.79
Fe	152.00	0.27	70.25	59.84
Pt	168.00	0.38	192.75	60.87
Ni	219.00	0.31	136.38	83.59

## Data Availability

Not applicable.
